# Pre-Treatment CD44 and CD47 Expression and Pathological Complete Response After Neoadjuvant Therapy in Breast Cancer: An Exploratory Single-Center Study

**DOI:** 10.3390/ijms27219671

**Published:** 2026-10-28

**Authors:** Hatice Asoglu, Esra Asarkaya, Abdurrahman Aykut, Gunes Dorukhan Cavusoglu, Yasemin Aydinalp Camadan, Mehmet Mutlu Kidi, Sedat Biter, Sendag Yaslikaya, Ramazan Asoglu, Tugba Toyran, Melek Ergin, Ertugrul Bayram, Ismail Oguz Kara, Berksoy Sahin, Semra Paydas, Tolga Koseci

**Affiliations:** 1 Department of Medical Oncology, Faculty of Medicine, Çukurova University, Adana 01330, Türkiye; 2 Department of Medical Oncology, Seyhan State Hospital, Adana 01150, Türkiye; 3 Department of Medical Oncology, Necip Fazil City Hospital, Kahramanmaras 46080, Türkiye; 4 Department of Medical Oncology, Giresun Training and Research Hospital, Giresun 28100, Türkiye; 5 Department of Cardiology, Yalova State Hospital, Yalova Provincial Health Directorate, Yalova 77200, Türkiye; 6 Department of Pathology, Faculty of Medicine, Çukurova University, Adana 01330, Türkiye

**Keywords:** breast cancer, neoadjuvant therapy, pathological complete response, CD44, CD47, cancer stem cells, immunohistochemistry

## Abstract

CD44, a stem cell-associated adhesion molecule, and CD47, an anti-phagocytic checkpoint, have been implicated in breast cancer chemoresistance, but their relationship to neoadjuvant response is poorly defined. We assessed both markers by immunohistochemistry in pre-treatment core biopsies from 43 women treated with neoadjuvant therapy at a single center. Total pathological complete response (pCR; ypT0/is ypN0), designated the primary endpoint during peer review, occurred in 12 patients (27.9%), and breast pCR (ypT0/is) in 14 (32.6%). Total pCR rates were 18.2% in CD44-positive and 38.1% in CD44-negative tumors (odds ratio, 0.36; 95% CI, 0.09–1.46; Fisher exact *p* = 0.185). CD44 positivity was concentrated in rarely responding hormone receptor-positive/HER2-negative tumors, and stratification by subtype moved the odds ratio toward the null (0.62; exact 95% CI, 0.07–5.25). For CD47, the odds ratio per one-point increase in immunoreactivity score was 0.80 (95% CI, 0.54–1.19; *p* = 0.264), but immunoreactivity was low (only two tumors reached the prespecified high-expression threshold) and possibly technically compromised, so these findings are inconclusive. No statistically detectable association was observed between either marker and total or breast pCR; treatment could not be adjusted for, and only very large effects were detectable. These exploratory data do not provide sufficient evidence to support CD44 or CD47 as response markers.

## 1. Introduction

Breast cancer is the most commonly diagnosed cancer among women worldwide. Global estimates for 2022 indicate approximately 2.3 million new cases and 666,000 deaths, corresponding to 11.6% of all cancer diagnoses [1]. A substantial proportion of patients with locally advanced or biologically aggressive disease now receive neoadjuvant systemic therapy before definitive surgery.

Neoadjuvant treatment permits direct assessment of tumor sensitivity in vivo, and the pathological response observed at surgery carries prognostic information. In the CTNeoBC pooled analysis of 12 trials and 11,955 patients, pathological complete response (pCR) defined as ypT0/is ypN0 was associated with improved event-free survival (hazard ratio, 0.48; 95% CI, 0.43–0.54) and overall survival (hazard ratio, 0.36; 95% CI, 0.31–0.42), although pCR was not established as a surrogate endpoint at the trial level [2]. A subsequent meta-analysis confirmed this individual-level association, particularly in triple-negative and HER2-positive disease [3]. Definitions of pCR have nonetheless differed between trials, and standardized endpoint definitions for the neoadjuvant setting, including a preferred composite definition of pCR, have since been proposed [4].

Only a minority of patients achieve pCR, and the response rate differs markedly between molecular subtypes [2,3]. Identifying tumors unlikely to respond before treatment begins therefore remains clinically relevant, and several tissue-based markers, assessed by immunohistochemistry or flow cytometry, have been examined for this purpose with inconsistent results [5,6]. Markers associated with the cancer stem cell phenotype are biologically plausible candidates, because cells that survive cytotoxic therapy are thought to be enriched for stem-like properties.

The cancer stem cell model in breast cancer originated with the observation that a CD44-positive, CD24-negative/low subpopulation was capable of initiating tumors in immunodeficient mice at low cell numbers [7]. CD44 is a transmembrane glycoprotein that mediates hyaluronan binding, adhesion, and signaling, and it contributes to invasion, metastasis, and therapeutic resistance [8]. Epithelial–mesenchymal transition generates cells with stem-like features and a CD44-high phenotype, which links CD44 expression to a state rather than to a fixed lineage [9], and isoform switching within the CD44 gene has been shown to determine that state [10]. CD44 expression is enriched in basal-like tumors [11], and the CD44-positive/CD24-negative phenotype has been associated with poorer outcomes in a meta-analysis of breast cancer cohorts [12]. Because CD44 is expressed well beyond the stem-like compartment and as multiple isoforms, CD44 immunoreactivity alone is an incomplete surrogate for this phenotype [8,10].

Data relating CD44 to neoadjuvant chemotherapy response are less consistent. Low CD44 expression together with high TFAP2C has been reported to accompany a higher pCR rate [13], whereas pre-treatment expression of the CD44v9 isoform in triple-negative disease was not associated with pCR, although it predicted shorter distant metastasis-free survival [14]. Studies of other stem cell-associated markers have yielded mixed results. ALDH1 expression has been linked to resistance to sequential taxane- and anthracycline-based treatment and was increased in residual tumor after chemotherapy, whereas the proportion of CD44-positive/CD24-negative cells was neither associated with pCR nor changed by treatment in the same cohort [15]. CD44 transcript levels have been reported to fall after neoadjuvant therapy [16], while immunohistochemically defined CD44 positivity did not change significantly after treatment and was more frequent in tumors with poor pathological response [17].

CD47 is of interest for a different reason. It is a widely expressed transmembrane protein that engages signal regulatory protein alpha on macrophages and transmits an anti-phagocytic signal, thereby limiting innate immune clearance of tumor cells [18]. In breast cancer cells, hypoxia-inducible factor 1 has been shown to increase CD47 expression, promoting evasion of phagocytosis and maintenance of the cancer stem cell population [19], and a function-blocking CD47 antibody suppressed stem cell and epidermal growth factor signaling in preclinical models [20]. High CD47 expression has been associated with poorer survival across solid tumors in a meta-analysis [21], and in breast cancer it has been examined in relation to tumor-associated macrophages, tumor-infiltrating lymphocytes, and molecular subtype [22,23,24,25]. Transcriptomic analyses indicate that genes co-expressed with CD47 in breast tumors define an immune signature associated with favorable outcome and T-cell activation, particularly in basal-like and HER2-positive disease [26]. CD44 and CD47 may also converge in the hypoxic tumor microenvironment: CD44 is itself a target of hypoxia-inducible factor 1, and CD47 and CD44 transcript levels are correlated in primary breast cancers [19], while CD44 has been implicated in the drug resistance and immune evasion of breast cancer stem cells [27].

Despite this background, the relationship between CD44 and CD47 expression and response to neoadjuvant therapy is poorly defined, and CD47 in particular has been studied far less in this setting than its biological relevance would suggest. We therefore examined CD44 and CD47 expression by immunohistochemistry in pre-treatment core needle biopsies from patients with invasive breast carcinoma treated with neoadjuvant systemic therapy, to assess whether pre-treatment expression is associated with pathological complete response, defined primarily by the composite ypT0/is ypN0 endpoint. Because both response and marker expression may vary by molecular subtype, we also examined the extent to which any crude association reflected the subtype composition of the cohort.

## 2. Results

### 2.1. Study Cohort

**Figure 1:**
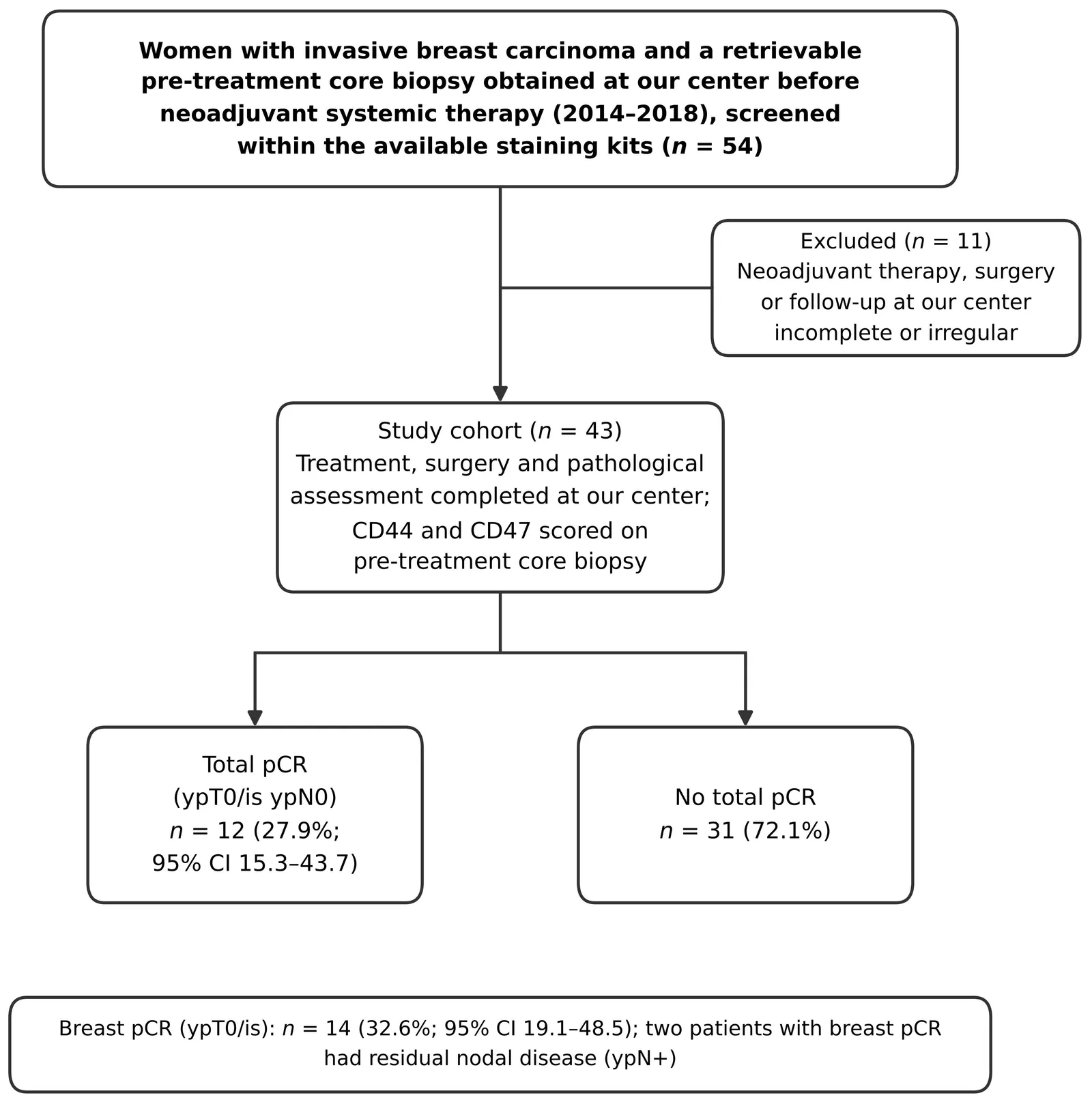
Flow of patients through the study. pCR, pathological complete response; total pCR, ypT0/is ypN0; breast pCR, ypT0/is; CI, confidence interval.

Of 54 patients screened, 11 (20.4%) were excluded because neoadjuvant therapy, surgery, or follow-up at our center was incomplete or irregular, and 43 formed the study cohort (Figure 1). Clinicopathological characteristics are summarized in Table 1. Twenty-two tumors (51.2%) were HR-positive/HER2-negative, 16 (37.2%) were HER2-positive (11 HR-positive and 5 HR-negative), and 5 (11.6%) were triple-negative; 40 patients (93.0%) were clinically node-positive at diagnosis. Neoadjuvant therapy included a taxane in 41 patients (95.3%), an anthracycline in 34 (79.1%), and a platinum agent in 11 (25.6%); all 16 patients with HER2-positive disease received trastuzumab, and none received pertuzumab.

### 2.2. Pathological Response

Total pCR (ypT0/is ypN0) was achieved in 12 of 43 patients (27.9%; 95% CI, 15.3–43.7) and breast pCR (ypT0/is) in 14 (32.6%; 95% CI, 19.1–48.5). The two endpoints were discordant in two patients, both with HER2-positive disease, who had no residual invasive carcinoma in the breast but residual nodal disease. Total pCR was achieved in 1 of 22 patients (4.5%) with HR-positive/HER2-negative disease, 6 of 16 (37.5%) with HER2-positive disease, and all 5 with triple-negative disease.

### 2.3. Pre-Treatment CD44 and CD47 Expression

The CD44 score was 0 in 21 patients (48.8%), 1 in 9 (20.9%), 2 in 12 (27.9%), and 3 in 1 (2.3%). CD44 was therefore positive in 22 patients (51.2%).

The CD47 immunoreactivity score (IRS) was 0 in 23 patients (53.5%), 1 in 7 (16.3%), 2 in 4 (9.3%), 4 in 5 (11.6%), 6 in 2 (4.7%), and 8 in 2 (4.7%). Any detectable CD47 expression was present in 20 tumors (46.5%), an IRS of 4 or higher in 9 (20.9%), and the prespecified threshold of IRS ≥ 7 was reached in 2 (4.7%). Non-neoplastic internal control staining (erythrocytes, lymphocytes, or endothelial cells) was confirmed in 38 of 43 sections (88.4%), including all 20 tumors with IRS > 0 and 18 of 23 tumors with IRS 0. In the remaining five IRS-0 cases, no internal control staining was detected, although the positive control on the same slide showed staining.

**Figure 2:**
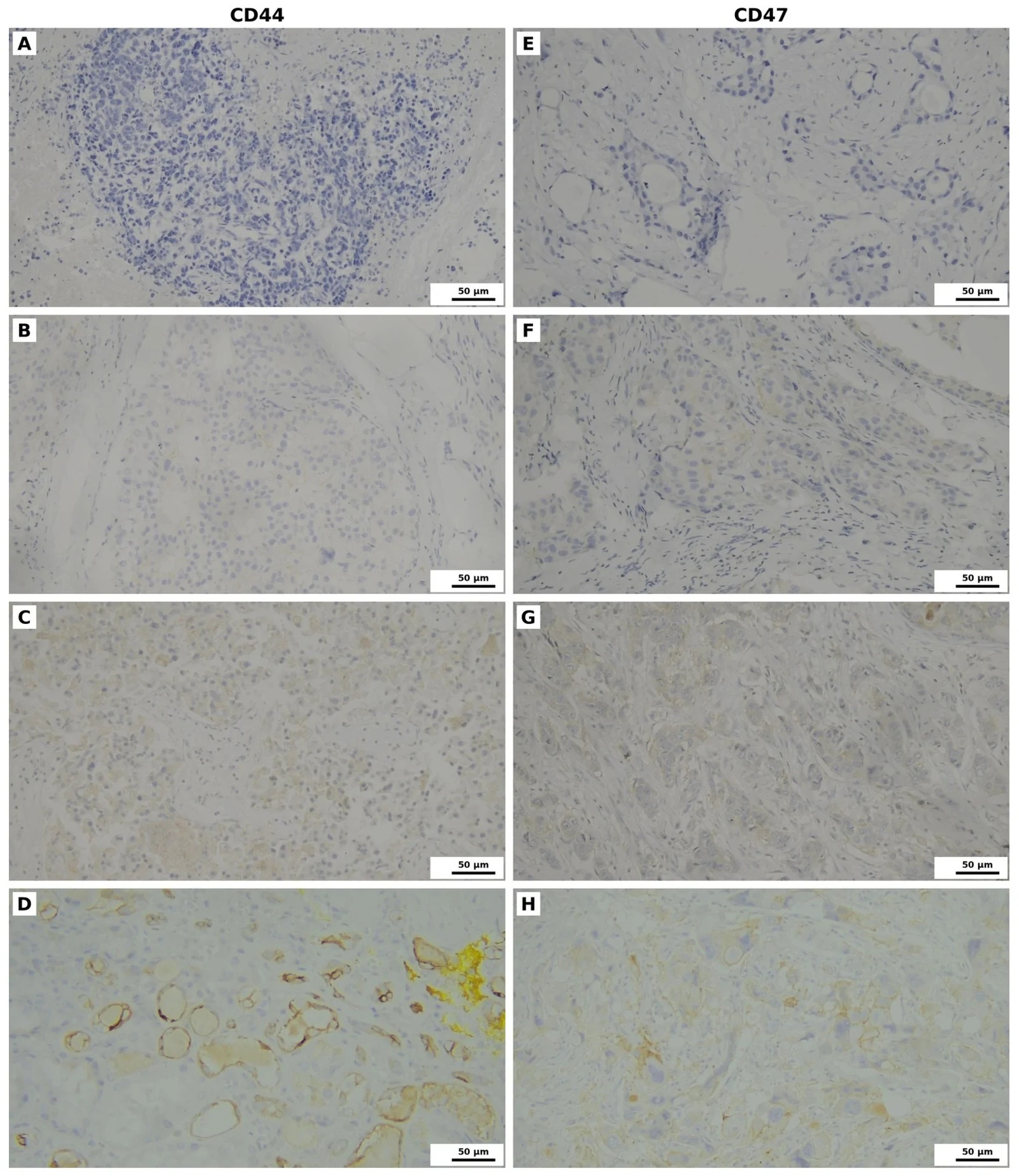
Representative pre-treatment immunohistochemical staining for each scoring category in invasive breast carcinoma (original uncropped fields; original magnification ×200). CD44: (**A**) score 0 (<10% of tumor cells stained); (**B**) score 1 (10–25%), weak membranous staining; (**C**) score 2 (25–50%), moderate membranous staining; (**D**) score 3 (>50%), strong membranous staining (intensity is described for illustration only and was not part of the CD44 score). CD47 (IRS = extent score 0–4 × intensity score 0–3): (**E**) IRS 0, with no staining of tumor cells; (**F**) weak membranous/cytoplasmic staining in a limited proportion of tumor cells (extent 2 × intensity 1; IRS 2); (**G**) more pronounced staining that did not reach the threshold for high expression (extent 3 × intensity 2; IRS 6); (**H**) widespread staining of moderate intensity in more than 80% of tumor cells (extent 4 × intensity 2; IRS 8), the highest score observed and representing high expression. IRS, immunoreactivity score. Scale bars, 50 µm.

Marker expression varied by subtype. CD44 was positive in 13 of 22 HR-positive/HER2-negative tumors (59.1%), 8 of 16 HER2-positive tumors (50.0%), and 1 of 5 triple-negative tumors; 7 of the 9 tumors with a CD47 IRS of 4 or higher were HR-positive/HER2-negative. Representative staining for each CD44 score and CD47 staining level is shown in Figure 2, and positive control staining is shown in Supplementary Figure S1.

### 2.4. Marker Expression and Total Pathological Complete Response

**Figure 3:**
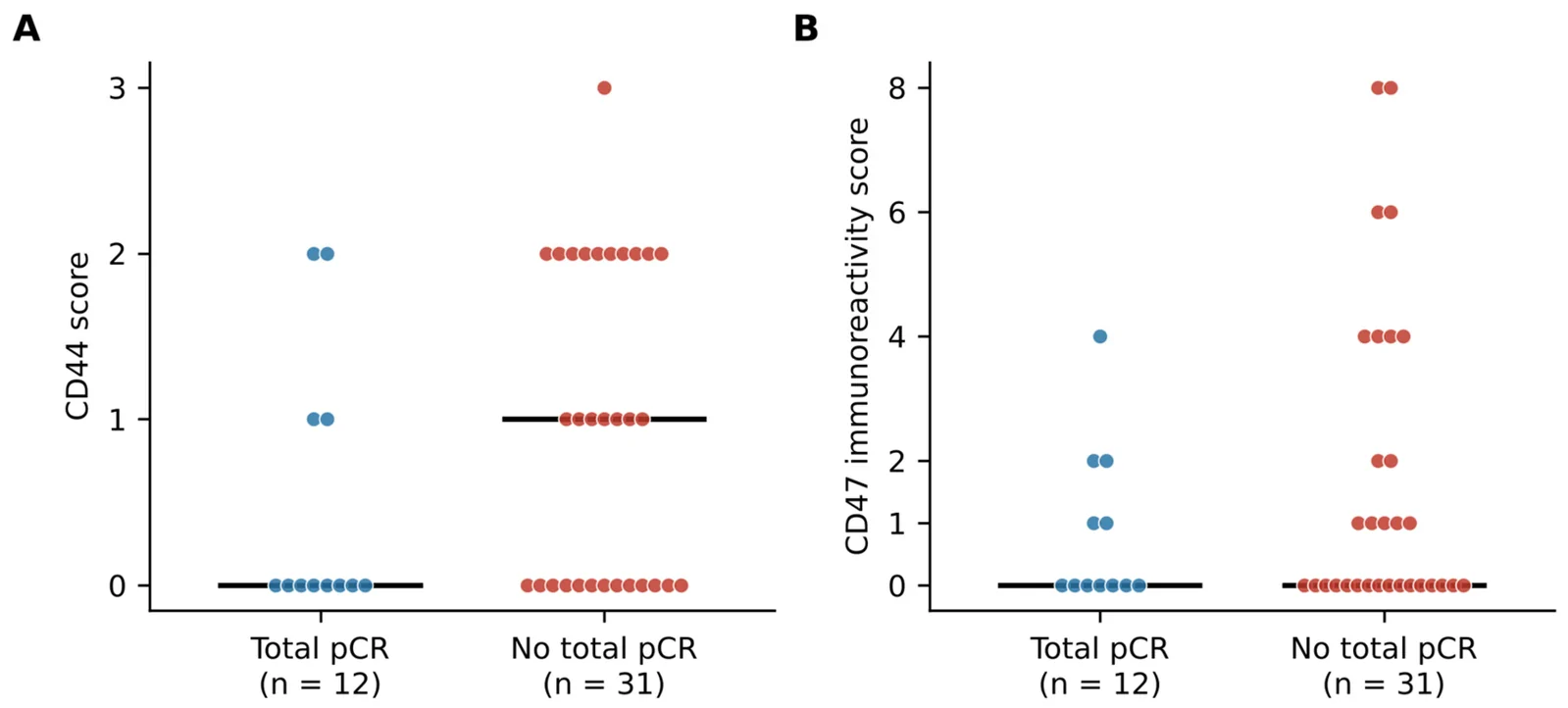
Individual pre-treatment marker scores according to total pathological complete response. (**A**) CD44 score; (**B**) CD47 immunoreactivity score. Each point represents one patient; points with the same score are spread horizontally to reduce overlap, and horizontal bars indicate medians. pCR, pathological complete response (ypT0/is ypN0).

Marker distributions according to total pCR are shown in Table 2, and individual scores in Figure 3. The median CD44 score was 0 (IQR, 0–1) in patients with total pCR and 1 (IQR, 0–2) in those without (*p* = 0.139; rank-biserial correlation, −0.27). Total pCR occurred in 4 of 22 patients with CD44-positive tumors (18.2%; 95% CI, 5.2–40.3) and in 8 of 21 with CD44-negative tumors (38.1%; 95% CI, 18.1–61.6) (*p* = 0.185).

The median CD47 IRS was 0 in both groups (IQR, 0–1.25 and 0–3, respectively; *p* = 0.488; rank-biserial correlation, −0.13). In post hoc categorizations, total pCR occurred in 5 of 20 patients with any CD47 expression (25.0%; 95% CI, 8.7–49.1) and in 7 of 23 without (30.4%; 95% CI, 13.2–52.9) (*p* = 0.745), and in 1 of 9 patients with an IRS of 4 or higher (11.1%; 95% CI, 0.3–48.2) and 11 of 34 with an IRS of 0–2 (32.4%; 95% CI, 17.4–50.5) (*p* = 0.405). Neither of the two patients whose tumors reached the prespecified threshold of IRS ≥ 7 achieved total pCR (*p* = 1.00).

In univariable logistic regression (Wald *p* values), the odds ratio for total pCR was 0.53 per one-point increase in CD44 score (95% CI, 0.23–1.24; *p* = 0.143) and 0.36 for CD44 positivity (95% CI, 0.09–1.46; *p* = 0.153). The corresponding estimates for CD47 were 0.80 per one-point increase in IRS (95% CI, 0.54–1.19; *p* = 0.264) and 0.76 for any expression (post hoc; 95% CI, 0.20–2.93; *p* = 0.692). When CD47 was modeled post hoc as a three-level variable, the odds ratios relative to IRS 0 were 1.31 for IRS 1–2 (95% CI, 0.29–5.95) and 0.29 for IRS ≥ 4 (95% CI, 0.03–2.74) (overall *p* = 0.377), indicating that the per-point estimate does not reflect a consistent trend. Firth penalized regression gave similar estimates for all models (Supplementary Table S1) and yielded a finite estimate for the prespecified threshold (IRS ≥ 7: odds ratio, 0.47; 95% CI, <0.01–6.37; *p* = 0.611).

### 2.5. Marker Expression and Breast Pathological Complete Response

Results for breast pCR were consistent with those for total pCR (Supplementary Table S2 and Figure S2). Breast pCR occurred in 5 of 22 patients with CD44-positive tumors (22.7%) and 9 of 21 with CD44-negative tumors (42.9%) (odds ratio, 0.39; 95% CI, 0.10–1.47; Fisher exact *p* = 0.203). The corresponding rates for CD47 were 6 of 20 (30.0%) with any expression (post hoc) and 8 of 23 (34.8%) without (odds ratio, 0.80; 95% CI, 0.22–2.90; Fisher exact *p* = 1.00).

### 2.6. Exploratory Analyses by Subtype and Treatment

No consistent association between CD44 status and total pCR was evident within molecular subtypes (Table 3), although the small subgroup sizes preclude reliable subtype-specific conclusions. Among HER2-positive tumors, total pCR occurred in 3 of 8 CD44-positive and 3 of 8 CD44-negative cases. Among HR-positive/HER2-negative tumors, the single total pCR occurred in a CD44-negative tumor (1 of 9 versus 0 of 13). All five triple-negative tumors achieved total pCR irrespective of marker status. The lower crude total pCR rate among CD44-positive tumors therefore coincided with an uneven distribution of CD44 positivity across subtypes. When the two informative strata (HR-positive/HER2-negative and HER2-positive) were combined, the subtype-stratified odds ratio for CD44 positivity was 0.62 (exact 95% CI, 0.07–5.25; exact conditional *p* = 0.668; Mantel–Haenszel estimate, 0.61), compared with a crude odds ratio of 0.36; the triple-negative stratum, in which all tumors achieved total pCR, was uninformative. For breast pCR, the stratified odds ratio was 0.64 (exact 95% CI, 0.08–5.03; *p* = 0.675; Mantel–Haenszel estimate, 0.63), compared with a crude odds ratio of 0.39.

Treatment categories largely followed subtype. All 22 patients with HR-positive/HER2-negative disease received an anthracycline (with a taxane in 21) and none received platinum, whereas platinum was given to 10 of 16 patients with HER2-positive and 1 of 5 with triple-negative disease. Tabulation by treatment category (Supplementary Table S3) therefore provided no information independent of subtype.

### 2.7. Minimum Detectable Effect

Given the observed group sizes and response rates, a two-sided Fisher exact test would have had 80% power to detect an association between CD44 status and total pCR only for odds ratios of 0.08 or lower or 6.9 or higher. For any CD47 expression, the corresponding threshold was an odds ratio of 6.7 or higher; in the direction of lower response, power did not reach 80% for any odds ratio (maximum, 75%). The corresponding thresholds for breast pCR were similar (CD44, ≤0.10 or ≥7.3; CD47, ≤ 0.02 or ≥7.1). These thresholds were calculated post hoc, at the observed allocation and baseline response rates.

### 2.8. Sensitivity Analyses

With a higher cut-off for CD44 positivity (score ≥ 2, i.e., >25% of tumor cells; post hoc), total pCR occurred in 2 of 13 tumors (15.4%; 95% CI, 1.9–45.4) and in 10 of 30 tumors with a score of 0–1 (33.3%; 95% CI, 17.3–52.8) (odds ratio, 0.36; 95% CI, 0.07–1.96; Fisher exact *p* = 0.290), and breast pCR in 3 of 13 (23.1%) and 11 of 30 (36.7%), respectively (odds ratio, 0.52; 95% CI, 0.12–2.30; Fisher exact *p* = 0.491) (Supplementary Table S4). After exclusion of the five tumors with IRS 0 in which no internal control element was stained, total pCR occurred in 5 of 20 tumors with any CD47 expression (25.0%) and in 6 of 18 without (33.3%) (Fisher exact *p* = 0.724), and the odds ratio per one-point increase in IRS was 0.77 (95% CI, 0.51–1.17; *p* = 0.220); results for IRS ≥ 4 and for breast pCR were similarly unchanged (Supplementary Table S5).

## 3. Discussion

In this exploratory single-center cohort, no statistically detectable association was observed between pre-treatment CD44 or CD47 expression in the diagnostic core biopsy and total pCR (ypT0/is ypN0) or breast pCR. Total pCR was numerically less frequent among CD44-positive tumors (18.2% versus 38.1%), but no consistent association between CD44 status and total pCR was evident within molecular subtypes, although the small subgroup sizes preclude reliable subtype-specific conclusions. Among HER2-positive tumors, total pCR rates were identical according to CD44 status. CD44 positivity was concentrated in HR-positive/HER2-negative tumors, in which only one total pCR occurred. With 12 events, the study had 80% power only for odds ratios below approximately 0.1 or above approximately 7 for CD44, and only above approximately 7 for CD47, so moderate associations in either direction cannot be excluded.

The principal contribution of this study is the subtype-stratified analysis, which indicates that the crude CD44 estimate is confounded by subtype. pCR rates differ several-fold between subtypes [2,3], and in this cohort total pCR ranged from 4.5% in HR-positive/HER2-negative to 100% in triple-negative disease. Any marker whose prevalence varies by subtype may therefore show a crude association with response, regardless of its biological role. CD44 expression is generally enriched in basal-like tumors [11], whereas CD44 positivity here was most frequent in HR-positive/HER2-negative tumors; with five triple-negative tumors, this may reflect sampling variation. In either case, the crude CD44 difference cannot be taken as evidence of chemoresistance. Stratification by subtype moved the odds ratio for CD44 positivity from 0.36 to 0.62, which quantifies this confounding, although the confidence interval remained wide (0.07–5.25). A recent study confined to 256 patients with triple-negative disease found that a composite CD44-high/CD24-low/ALDH1-positive phenotype, present in 10% of tumors, was associated with a lower pCR rate after adjustment for clinical factors [28]. A design restricted to a single subtype and using a composite phenotype avoids the confounding that limits the present analysis.

Previous studies of CD44 alone have produced inconsistent results. Low CD44 expression combined with high TFAP2C was associated with a higher pCR rate in 23 patients, using a threshold of 80% stained cells rather than the 10% used here [13]. The proportion of CD44-positive/CD24-negative cells was not associated with pCR in 108 patients [15], and pre-treatment CD44 immunohistochemistry was not associated with Miller–Payne grade in 91 patients [5]. By contrast, CD44 positivity decreased from poor to near-complete responders in a series of 92 patients [17]. None of these studies reported subtype-adjusted estimates, and differences in antibody clone, threshold, and cohort composition may account for the discrepancies. Pan-CD44 antibodies also do not distinguish the standard and variant isoforms, which have opposing associations with the stem cell state [10], so CD44 immunoreactivity should not be equated with the cancer stem cell phenotype. CD44 may also be more relevant to recurrence than to immediate response. CD44v9 expression predicted distant metastasis but not pCR in triple-negative disease [14], and CD44 upregulation in residual triple-negative tumors was associated with early recurrence in the MIRINAE trial [29]. The present study had no follow-up and cannot address this possibility.

No statistically detectable association was observed between CD47 immunoreactivity and total or breast pCR under any of the categorizations examined. The prespecified threshold of IRS ≥ 7 was reached by only two tumors and was uninformative. Excluding the five IRS-0 tumors without internal control staining did not change these results. Because CD47 immunoreactivity was low and possibly technically compromised, these findings are inconclusive rather than negative. The lower response rate among the nine tumors with an IRS of 4 or higher should not be interpreted, since seven of them were HR-positive/HER2-negative. Most CD47 studies in breast cancer have examined resected tumors in relation to macrophages, lymphocytes, subtype, and survival rather than response [22,23,24,25,30]. In a recent analysis of resected breast cancers, CD47 expression correlated with a phagocytic macrophage gene signature [31]. The macrophage context would therefore need to be characterized alongside CD47 in future studies of response [22,23,24,32].

A more specific rationale exists in HER2-positive disease. In preclinical models, the antitumor activity of trastuzumab depended largely on macrophage-mediated antibody-dependent cellular phagocytosis, which CD47 blockade enhanced [33,34]. Tumor CD47 expression was inversely associated with survival in HER2-positive disease [33], and the cytotoxic payload of trastuzumab deruxtecan increased tumor CD47 expression in experimental models [35]. Tumor CD47 could therefore modify response specifically to anti-HER2 antibodies. With 16 HER2-positive patients, the present cohort was too small to examine this question, which would require a HER2-positive series with uniform, documented anti-HER2 therapy. Clinical development of CD47-targeted agents has produced mixed results [18,36], and development of magrolimab has been discontinued [37].

Pathological response now guides post-neoadjuvant therapy [38,39], and in a recent series of 607 patients, pCR was associated with better outcomes across all survival endpoints [40]; a marker identifying tumors unlikely to respond could therefore inform the choice of neoadjuvant regimen. However, patients in this cohort were treated before neoadjuvant pembrolizumab became standard for triple-negative disease [41], and none with HER2-positive disease received pertuzumab, so any marker association would require prospective validation with contemporary regimens.

### 3.1. Limitations

This study has several limitations.

First, with 43 patients and 12 total pCR events, the study had adequate power only for very large associations, with minimum detectable odds ratios of approximately 0.1 or 7 for CD44 and approximately 7 for CD47. The absence of a statistically significant association should therefore be interpreted as an absence of evidence rather than as evidence of no association.

Second, adjustment for confounding was limited: a subtype-stratified estimate was feasible, but multivariable modeling was not, and treatment could not be adjusted for. Subtype was strongly associated with both response and marker distribution, and treatment categories closely followed subtype, so the crude estimates reflect cohort composition as much as marker biology. Doses and numbers of cycles were not available, no patient with HER2-positive disease received pertuzumab, and treatment in 2014–2018 differed from current practice.

Third, the study was retrospective and single-center and relied on archival material. The cohort represents a small subset of the patients treated with neoadjuvant therapy at our center during the study period: consecutive patients were included in chronological order only until the available staining kits were exhausted, and 11 of 54 screened patients (20.4%) were excluded because neoadjuvant therapy, surgery, or follow-up at our center was incomplete or irregular. The representativeness of the cohort therefore cannot be established, and selection bias cannot be excluded. The cohort included only five triple-negative tumors and a high proportion of clinically node-positive disease (93.0%), and the total pCR rate of 4.5% in HR-positive/HER2-negative disease was lower than reported for this subtype in pooled trial data [2]; these features limit generalizability.

Fourth, the distribution of CD47 immunoreactivity was narrow. No staining was recorded in 23 tumors (53.5%), and only two reached IRS ≥ 7, whereas surgical series using the same score reported high expression in 44.5% to 64.5% of tumors [22,24,25], including one using the same clone [25]. Antigen retrieval at pH 9, used in another series [32], did not improve staining during optimization, but the use of core biopsies rather than resection specimens and block age (approximately 4 to 9 years at the time of staining) may have reduced immunoreactivity. The positive control and non-neoplastic cells stained only faintly in some sections, and in 5 of the 23 tumors with IRS 0 no internal control element was stained, although the positive control on the same slide was; false-negative CD47 results therefore cannot be excluded in these tumors, although their exclusion did not change the results. The slides were scored jointly by two pathologists rather than independently, so interobserver agreement could not be assessed, which is particularly relevant for a semiquantitative score such as the CD47 IRS. The post hoc CD47 categorizations are exploratory.

Fifth, CD44 was assessed with a pan-CD44 antibody, by staining extent only, and without ALDH1 or isoform-specific analysis [5,7,12,15]. CD24 data were not included because the clone used has not been validated for human tissue (Section 4.4), so the CD44-positive/CD24-negative phenotype was not assessed. The results therefore apply to CD44 immunoreactivity and not to the cancer stem cell phenotype.

Finally, long-term survival data were not collected for this study. The findings concern association with pathological response only and do not address the prognostic value of either marker. No multiplicity adjustment was applied, and all analyses are exploratory.

### 3.2. Strengths

Slides were jointly reviewed by two pathologists blinded to response, and a consensus score was assigned. Post-treatment nodal status was available for every patient, allowing use of the composite endpoint recommended by NeoSTEEP [4]. Exact confidence intervals and penalized regression appropriate to the sample size were used, individual and subtype-stratified data are reported, and prespecified and post hoc analyses are distinguished throughout.

## 4. Materials and Methods

### 4.1. Study Design and Patients

This single-center retrospective study was conducted at the Department of Medical Oncology and the Department of Pathology, Faculty of Medicine, Çukurova University, Adana, Türkiye. The broader institutional project from which this study derives was approved by the Non-Interventional Clinical Research Ethics Committee of the Faculty of Medicine, Çukurova University (meeting no. 121, decision no. 75; 8 April 2022), and the present retrospective analysis was approved by the same committee, now the Research Ethics Committee of the Faculty of Medicine, Çukurova University (meeting no. 168, decision no. 60; 10 July 2026). The study was performed in accordance with the Declaration of Helsinki and is reported in accordance with the REMARK recommendations for tumor marker studies [42].

Women with histologically confirmed invasive breast carcinoma whose pre-treatment core needle biopsy, obtained at our center between 2014 and 2018 before neoadjuvant systemic therapy, was retrievable from the institutional pathology archive were considered for inclusion. These patients were identified in chronological order from the archive, and consecutive patients whose biopsy block could be retrieved and contained sufficient tumor tissue were screened until the number of available staining kits was reached (n = 54). Pathological response was not known at the time of block selection. The total number of patients treated with neoadjuvant therapy at our center during the study period could not be reliably determined from institutional records. Patients were eligible when neoadjuvant therapy, surgery, and pathological assessment had all been completed at our center, with regular follow-up from the start of neoadjuvant therapy to surgery and with complete clinical and pathological records. Of the 54 screened patients, 11 (20.4%) were excluded because neoadjuvant therapy, surgery, or follow-up at our center was incomplete or irregular, and the remaining 43 formed the study cohort (Figure 1). All records were cross-checked against the original accession registers of the pathology archive, and one transcription error in a biopsy accession number was identified and corrected.

### 4.2. Clinicopathological Variables

Age, menopausal status, histological type and grade, tumor laterality, Ki-67 index, hormone receptor and HER2 status, and clinical tumor and nodal stage at diagnosis were abstracted from the electronic medical record and the original diagnostic pathology reports; no receptor testing was repeated for this study. Histological grade was assigned according to the Nottingham grading system, and clinical stage according to the American Joint Committee on Cancer (AJCC) Cancer Staging Manual, 8th edition. The Ki-67 index was recorded as the percentage of positively stained tumor nuclei given in the diagnostic report. No Ki-67 threshold was applied in the diagnostic reports or in the present analysis. Variables that could not be retrieved are reported as unknown in Table 1.

Hormone receptor positivity was defined as estrogen and/or progesterone receptor staining in at least 1% of tumor nuclei. Tumors with estrogen receptor (ER) staining in 1–10% of nuclei, now reported as ER-low positive [43], were classified as hormone receptor-positive; two tumors fell into this category, one HER2-negative without pCR and one HER2-positive with total pCR. HER2 positivity was defined as an immunohistochemical score of 3+, or 2+ with amplification on in situ hybridization. Tumors were classified as HR-positive/HER2-negative, HER2-positive irrespective of hormone receptor status, or triple-negative (estrogen and progesterone receptor staining in <1% of nuclei and HER2-negative).

### 4.3. Neoadjuvant Treatment and Assessment of Pathological Response

Neoadjuvant regimens were administered according to institutional practice at the time of treatment. Exposure to anthracyclines, taxanes, platinum agents, and trastuzumab was recorded for each patient from the hospital information system. No patient received pertuzumab or neoadjuvant endocrine therapy, and doses and numbers of cycles were not analyzed.

Surgical specimens were examined by breast pathologists as part of routine diagnostic workup, and post-treatment axillary nodal status was obtained from the surgical pathology reports of sentinel lymph node biopsy and/or axillary lymph node dissection. The primary endpoint was total pathological complete response (total pCR), defined as the absence of residual invasive carcinoma in the breast and axillary lymph nodes (ypT0/is ypN0), in accordance with the NeoSTEEP consensus [4]. The secondary endpoint was breast pathological complete response (breast pCR), defined as the absence of residual invasive carcinoma in the breast irrespective of nodal status (ypT0/is). Residual ductal carcinoma in situ was permitted in both definitions. Total pCR was designated the primary endpoint during revision, after post-treatment nodal status had been retrieved for all patients; breast pCR, the endpoint of the original analysis, was retained as the secondary endpoint.

### 4.4. Immunohistochemistry

Hematoxylin and eosin-stained slides of all pre-treatment core needle biopsies were reviewed, and one representative paraffin block was selected for each on the basis of adequate tumor content, adequacy of fixation, extent of necrosis, presence of adjacent non-neoplastic breast tissue, and absence of artifacts such as cautery or folding.

Sections of 4 µm were cut from the selected blocks and mounted on poly-L-lysine-coated slides. All biopsy specimens had been fixed in 10% neutral buffered formalin for 12–24 h according to the institutional standard protocol (individual fixation times were not recorded) and embedded in paraffin; sections were stained within 48 h of cutting, and CD47 immunohistochemistry was performed for the entire cohort in a single batch in January 2023, when the blocks were approximately 4 to 9 years old. After deparaffinization and rehydration, antigen retrieval was performed in citrate buffer (pH 6.0) using microwave heating, and endogenous peroxidase activity was quenched with 3% hydrogen peroxide. Sections were then incubated with the primary antibodies listed in Table 4; the anti-CD47 antibody (clone B6H12, previously applied to formalin-fixed, paraffin-embedded breast carcinoma [25]) was applied overnight at 4 °C. This clone is recommended by the vendor for immunohistochemistry on paraffin-embedded sections; before the study, dilutions of 1:100, 1:150, and 1:200 were tested on representative sections, and 1:150 was selected as giving the best balance between specific staining and background. Antigen retrieval at pH 9 was also tested and did not improve CD47 staining. Immunoreactivity was visualized using the EnVision detection system (Dako, Glostrup, Denmark), followed by hematoxylin counterstaining. Sections processed without the primary antibody served as negative controls, and each slide carried at least two cases together with a positive control section; normal breast tissue served as the positive control for CD44 and prostate tissue for CD47 (Table 4). Images were captured with the same digital camera system mounted on an Olympus CX33 microscope (Olympus, Tokyo, Japan), using a ×20 objective (original magnification ×200) and identical acquisition settings. Scale bars were drawn from a calibration of 0.299 µm per pixel, obtained from a calibrated reference image acquired with the same settings. Representative images of each CD44 and CD47 scoring category are shown in Figure 2, and images of the CD44 and CD47 positive controls are shown in Supplementary Figure S1.

CD24 and CD133 were also stained on sections cut from the same blocks as part of a broader institutional project, and neither has been published elsewhere. CD24 data were not included in the present biomarker analysis because the anti-CD24 clone used (M1/69) has not been validated for human tissue, and CD133 results are outside the scope of the present analysis. Residual invasive carcinoma in the surgical specimens of the 29 patients without breast pCR was also stained for CD44 and CD47 using the same protocol; these post-treatment data are outside the scope of the present analysis, which is restricted to pre-treatment expression. The present analysis is therefore one of several from the same staining set; the CD133 and post-treatment data are not planned for separate publication.

### 4.5. Evaluation of Immunohistochemical Staining

All slides were evaluated jointly by two pathologists at ×10, ×20, and ×40 magnification, with the entire tumor area of each section assessed, and a single consensus score was assigned to each case. Both pathologists were blinded to the clinical data, including response status. Because scoring was performed jointly, interobserver agreement could not be assessed. All 43 CD47-stained sections were additionally re-reviewed for staining of non-neoplastic internal control elements (erythrocytes, lymphocytes, and endothelial cells).

For CD44, membranous staining of tumor cells was regarded as specific [44,45]. The proportion of stained tumor cells was scored as 0 (<10%), 1 (10–25%), 2 (25–50%), or 3 (>50%), and scores of 1 to 3 were regarded as positive, as described for the same clone (156-3C11) [45]. Staining intensity was not incorporated into the CD44 score. Normal breast tissue was used as the reference for CD44 staining.

For CD47, staining extent and staining intensity were recorded separately, following the immunoreactivity score applied in previous breast cancer studies [22,24,25]. Extent was scored as 0 (no staining), 1 (1–10%), 2 (11–50%), 3 (51–80%), or 4 (81–100%) of tumor cells. Intensity was scored as 0 (absent), 1 (weak), 2 (moderate), or 3 (strong). An immunoreactivity score (IRS) ranging from 0 to 12 was obtained by multiplying extent by intensity. A threshold of IRS ≥ 7 was adopted from these studies to define high CD47 expression before any comparison with pathological response; it was not derived from the present data.

### 4.6. Statistical Analysis

Age is presented as mean ± standard deviation; Ki-67 and ordinal marker scores as median with interquartile range; and categorical variables as counts with percentages. Proportions are reported with Clopper–Pearson exact 95% confidence intervals. Marker scores were compared between patients with and without pCR using the Mann–Whitney U test with continuity correction (asymptotic two-sided *p* values, corrected for ties), with the rank-biserial correlation as the effect size (calculated from the U statistic, with negative values indicating lower scores in patients with pCR), and dichotomized marker categories were compared using the Fisher exact test. *p* values for dichotomized markers are Fisher exact *p* values throughout, and regression-based *p* values are labeled as such. All analyses were performed for the primary endpoint (total pCR) and repeated for the secondary endpoint (breast pCR).

Because only two tumors reached the prespecified CD47 threshold, CD47 was analyzed primarily as an ordinal variable. Three further categorizations were defined post hoc: any detectable expression (IRS > 0); IRS ≥ 4 versus 0–2, as a sensitivity analysis at an intermediate threshold (no tumor had an IRS of 3, so thresholds of 3 and 4 are equivalent in this cohort); and a three-level categorical variable (IRS 0, 1–2, and ≥ 4), used to examine the linearity assumption of the per-point model.

Univariable logistic regression was used to estimate odds ratios (ORs) with Wald 95% confidence intervals for CD44 score (per point), CD44 status, CD47 IRS (per point), and each CD47 categorization; the overall association of the three-level CD47 variable was assessed with a likelihood ratio test. As a sensitivity analysis, all models were refitted using Firth’s penalized likelihood logistic regression, which reduces small-sample bias and provides finite estimates under separation, with profile penalized likelihood confidence intervals and penalized likelihood ratio tests [46]. Given 12 total pCR events, multivariable modeling was not performed. Marker distributions and pCR rates were tabulated descriptively by molecular subtype and by neoadjuvant treatment category, without formal testing. Post hoc, the association between CD44 status and pCR was also estimated with stratification by subtype, combining the HR-positive/HER2-negative and HER2-positive strata (the triple-negative stratum, in which all tumors achieved total pCR, was uninformative), using the Mantel–Haenszel estimator and exact conditional inference with exact 95% confidence intervals. Two further sensitivity analyses were defined post hoc: CD44 dichotomized at a higher cut-off (score ≥ 2), and the CD47 analyses repeated after exclusion of the five IRS-0 tumors in which no internal control element was stained.

To aid interpretation of non-significant results, the minimum detectable OR was calculated post hoc for each dichotomized marker. It was defined as the smallest OR, in either direction, at which a two-sided Fisher exact test at α = 0.05 would have 80% power, given the observed group sizes and the observed pCR rate in marker-negative tumors; power was computed exactly by enumeration of all binomial outcomes.

Histological grade and clinical T stage were missing in two patients each and are reported as unknown; neither variable was included in the models, and all other variables were complete. All tests were two-sided, and *p* < 0.05 was considered to indicate nominal statistical significance. No multiplicity adjustment was prespecified and none was applied; all analyses are exploratory. Statistical analyses were performed using IBM SPSS Statistics, version 25.0 (IBM Corp., Armonk, NY, USA) and R, version 4.6.0 (R Foundation for Statistical Computing, Vienna, Austria). Descriptive statistics, Fisher exact tests, Mann–Whitney U tests, and standard logistic regression were performed in SPSS. Firth penalized logistic regression was performed with the logistf package, version 1.26.1 [46]; effect sizes, exact confidence intervals, minimum detectable odds ratios, and the stratified and sensitivity analyses were calculated with the base functions of R.

## 5. Conclusions

In this exploratory single-center cohort, no statistically detectable association was observed between pre-treatment CD44 or CD47 immunoreactivity in the diagnostic core biopsy and total or breast pathological complete response. The lower crude response rate among CD44-positive tumors coincided with an uneven distribution of CD44 positivity across molecular subtypes, and no consistent association between CD44 status and total pCR was evident within molecular subtypes, although the small subgroup sizes preclude reliable subtype-specific conclusions. Stratification by subtype moved the odds ratio for CD44 positivity toward the null.

With 12 events, a narrow CD47 score distribution, only a stratified adjustment for subtype, and no adjustment for neoadjuvant treatment, which largely followed subtype and lacked regimen-level detail, the study was insufficient to evaluate moderate associations. CD47 immunoreactivity was low and possibly technically compromised, so the CD47 findings should be regarded as inconclusive rather than negative. Patients were treated in 2014–2018, before neoadjuvant pembrolizumab became standard for triple-negative disease and without pertuzumab for HER2-positive disease, so the findings may not apply to contemporary regimens. These data do not provide sufficient evidence to support either marker as a marker of response. Further evaluation would require larger, subtype-restricted cohorts with standardized scoring and documented contemporary treatment. For CD47, a HER2-positive series receiving uniform anti-HER2 therapy would be the most informative setting.

## Figures and Tables

**Table 1:** Clinicopathological characteristics of the study cohort according to total pathological complete response (*n* = 43).

Characteristic	All (*n* = 43)	Total pCR (*n* = 12)	No Total pCR (*n* = 31)
Age, years, mean ± SD	52.5 ± 11.6	54.8 ± 14.2	51.5 ± 10.6
Menopausal status			
Premenopausal	19 (44.2)	4 (33.3)	15 (48.4)
Postmenopausal	24 (55.8)	8 (66.7)	16 (51.6)
Histology			
Invasive ductal carcinoma	40 (93.0)	11 (91.7)	29 (93.5)
Other	3 (7.0)	1 (8.3)	2 (6.5)
Histological grade			
2	22 (51.2)	5 (41.7)	17 (54.8)
3	19 (44.2)	6 (50.0)	13 (41.9)
Unknown	2 (4.7)	1 (8.3)	1 (3.2)
Ki-67, %, median (IQR)	30 (20–45)	40 (20–55)	30 (12.5–40)
Tumor laterality			
Right	23 (53.5)	9 (75.0)	14 (45.2)
Left	20 (46.5)	3 (25.0)	17 (54.8)
Clinical T stage			
cT1	3 (7.0)	0 (0.0)	3 (9.7)
cT2	26 (60.5)	6 (50.0)	20 (64.5)
cT3	4 (9.3)	2 (16.7)	2 (6.5)
cT4	8 (18.6)	3 (25.0)	5 (16.1)
Unknown	2 (4.7)	1 (8.3)	1 (3.2)
Clinical N stage			
cN0	3 (7.0)	1 (8.3)	2 (6.5)
cN1	26 (60.5)	6 (50.0)	20 (64.5)
cN2	7 (16.3)	3 (25.0)	4 (12.9)
cN3	7 (16.3)	2 (16.7)	5 (16.1)
Subtype (HR/HER2) ^b^			
HR+/HER2−	22 (51.2)	1 (8.3)	21 (67.7)
HR+/HER2+	11 (25.6)	2 (16.7)	9 (29.0)
HR−/HER2+	5 (11.6)	4 (33.3)	1 (3.2)
Triple-negative	5 (11.6)	5 (41.7)	0 (0.0)
Neoadjuvant treatment ^a^			
Anthracycline	34 (79.1)	7 (58.3)	27 (87.1)
Taxane	41 (95.3)	12 (100.0)	29 (93.5)
Platinum	11 (25.6)	6 (50.0)	5 (16.1)
Trastuzumab	16 (37.2)	6 (50.0)	10 (32.3)

Values are n (%) unless otherwise stated; percentages are calculated within columns. ^a^ Categories are not mutually exclusive. ^b^ HR+/HER2+ and HR−/HER2+ tumors are combined as HER2-positive in subsequent analyses. Clinical stage according to the American Joint Committee on Cancer (AJCC) Cancer Staging Manual, 8th edition. The difference in tumor laterality between response groups has no known biological basis and is most likely due to chance. pCR, pathological complete response (ypT0/is ypN0); SD, standard deviation; IQR, interquartile range; HR, hormone receptor.

**Table 2:** Pre-treatment CD44 and CD47 expression according to total pathological complete response (*n* = 43).

	Total pCR (*n* = 12), *n* (%)	No Total pCR (*n* = 31), *n* (%)	Total pCR Rate, % (95% CI)	*p*
CD44 score, median (IQR)	0 (0–1)	1 (0–2)	—	0.139 ^a^
0	8 (66.7)	13 (41.9)	38.1 (18.1–61.6)	
1	2 (16.7)	7 (22.6)	22.2 (2.8–60.0)	
2	2 (16.7)	10 (32.3)	16.7 (2.1–48.4)	
3	0 (0.0)	1 (3.2)	0.0 (0.0–97.5)	
CD44 status				0.185 ^b^
Negative (score 0)	8 (66.7)	13 (41.9)	38.1 (18.1–61.6)	
Positive (score ≥ 1)	4 (33.3)	18 (58.1)	18.2 (5.2–40.3)	
CD47 IRS, median (IQR)	0 (0–1.25)	0 (0–3)	—	0.488 ^a^
0	7 (58.3)	16 (51.6)	30.4 (13.2–52.9)	
1	2 (16.7)	5 (16.1)	28.6 (3.7–71.0)	
2	2 (16.7)	2 (6.5)	50.0 (6.8–93.2)	
4	1 (8.3)	4 (12.9)	20.0 (0.5–71.6)	
6	0 (0.0)	2 (6.5)	0.0 (0.0–84.2)	
8	0 (0.0)	2 (6.5)	0.0 (0.0–84.2)	
CD47, any expression ^c^				0.745 ^b^
IRS 0	7 (58.3)	16 (51.6)	30.4 (13.2–52.9)	
IRS > 0	5 (41.7)	15 (48.4)	25.0 (8.7–49.1)	
CD47, sensitivity threshold ^c^				0.405 ^b^
IRS 0–2	11 (91.7)	23 (74.2)	32.4 (17.4–50.5)	
IRS ≥ 4	1 (8.3)	8 (25.8)	11.1 (0.3–48.2)	
CD47, prespecified threshold				1.00 ^b^
IRS 0–6	12 (100.0)	29 (93.5)	29.3 (16.1–45.5)	
IRS ≥ 7	0 (0.0)	2 (6.5)	0.0 (0.0–84.2)	

Percentages in the first two columns are calculated within response groups; total pCR rates are calculated within marker categories, with Clopper–Pearson exact confidence intervals. ^a^ Mann–Whitney U test. ^b^ Fisher exact test. ^c^ Defined post hoc. pCR, pathological complete response (ypT0/is ypN0); IRS, immunoreactivity score; IQR, interquartile range; CI, confidence interval.

**Table 3:** Marker expression and pathological complete response according to molecular subtype (*n* = 43).

	HR+/HER2− (*n* = 22)	HER2+ (*n* = 16)	Triple-Negative (*n* = 5)
Total pCR	1/22 (4.5)	6/16 (37.5)	5/5 (100.0)
Breast pCR	1/22 (4.5)	8/16 (50.0)	5/5 (100.0)
CD44 positive	13/22 (59.1)	8/16 (50.0)	1/5 (20.0)
CD47 IRS > 0	10/22 (45.5)	7/16 (43.8)	3/5 (60.0)
CD47 IRS ≥ 4	7/22 (31.8)	1/16 (6.3)	1/5 (20.0)
Total pCR by CD44 status			
CD44 negative	1/9	3/8	4/4
CD44 positive	0/13	3/8	1/1
Total pCR by CD47 status			
IRS 0	1/12	4/9	2/2
IRS > 0	0/10	2/7	3/3

Values are *n/N* (%) or *n/N*. Of the 16 HER2-positive tumors, 11 were HR-positive (total pCR, 2 of 11; breast pCR, 3 of 11) and 5 were HR-negative (total pCR, 4 of 5; breast pCR, 5 of 5). No statistical testing was performed because of the small number of patients in each subgroup. pCR, pathological complete response (total, ypT0/is ypN0; breast, ypT0/is); HR, hormone receptor; IRS, immunoreactivity score.

**Table 4:** Details of the immunohistochemical staining protocol.

Antibody	Clone	Source (Catalogue No.)	Dilution	Positive Control
CD44	156-3C11, monoclonal	Thermo Fisher/Lab Vision (MS-668-R7)	Ready to use	Normal breast tissue
CD47	B6H12, monoclonal	Santa Cruz Biotechnology (sc-12730)	1:150	Prostate tissue

## Data Availability

The patient-level data supporting the findings of this study are not publicly available because they derive from a small single-center cohort in which combinations of clinical and pathological variables could permit re-identification, and because public deposition of patient-level data is not covered by the ethics approval for this retrospective study. De-identified data are available from the corresponding author upon reasonable request, subject to approval by the Research Ethics Committee of the Faculty of Medicine, Çukurova University.

## Supplementary Materials

The following supporting information can be downloaded at: https://www.mdpi.com/article/10.3390/ijms27219671/s1.

## Abbreviations

AJCC, American Joint Committee on Cancer; CI, confidence interval; ER, estrogen receptor; HR, hormone receptor; IQR, interquartile range; IRS, immunoreactivity score; OR, odds ratio; pCR, pathological complete response; SD, standard deviation.
