# Supplementary material for: Pre-Treatment CD44 and CD47 Expression and Pathological Complete Response After Neoadjuvant Therapy in Breast Cancer: An Exploratory Single-Center Study
Source: Int J Mol Sci. 2026 Oct 28;27(21):9671. doi: 10.3390/ijms27219671 (PMC13763045; doi:10.3390/ijms27219671)
Supplement: Supplementary file 1 [file ijms-27-09671-s001.zip › ijms-4615057-supplementary.pdf]

Supplementary Materials

Pre-Treatment CD44 and CD47 Expression and Pathological Complete Response After Neoadjuvant Therapy in Breast Cancer: An Exploratory Single-Center Study

Contents

Figure S1. Positive control staining for CD44 and CD47.

Figure S2. Individual pre-treatment marker scores according to breast pathological complete response.

Table S1. Univariable logistic regression for total and breast pathological complete response: standard and Firth penalized estimates.

Table S2. Pre-treatment CD44 and CD47 expression according to breast pathological complete response.

Table S3. Pathological complete response and marker expression according to neoadjuvant treatment category.

Table S4. Sensitivity analysis of CD44 at a higher cut-off (score ≥ 2).

Table S5. Sensitivity analysis of CD47 after exclusion of the five tumors without internal control staining.

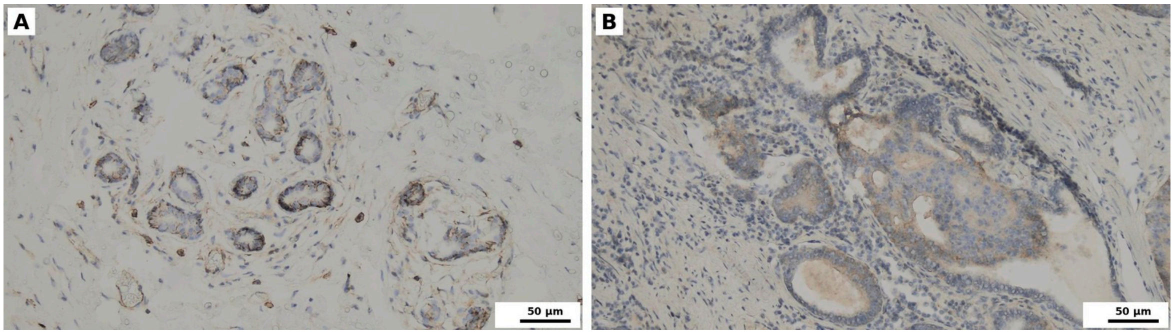

Figure S1. Positive control staining (original uncropped fields; original magnification ×200). (A) CD44, normal breast tissue, showing membranous staining of epithelial cells. (B) CD47, prostate tissue, showing cytoplasmic staining of glandular epithelium. Scale bars, 50 µm.

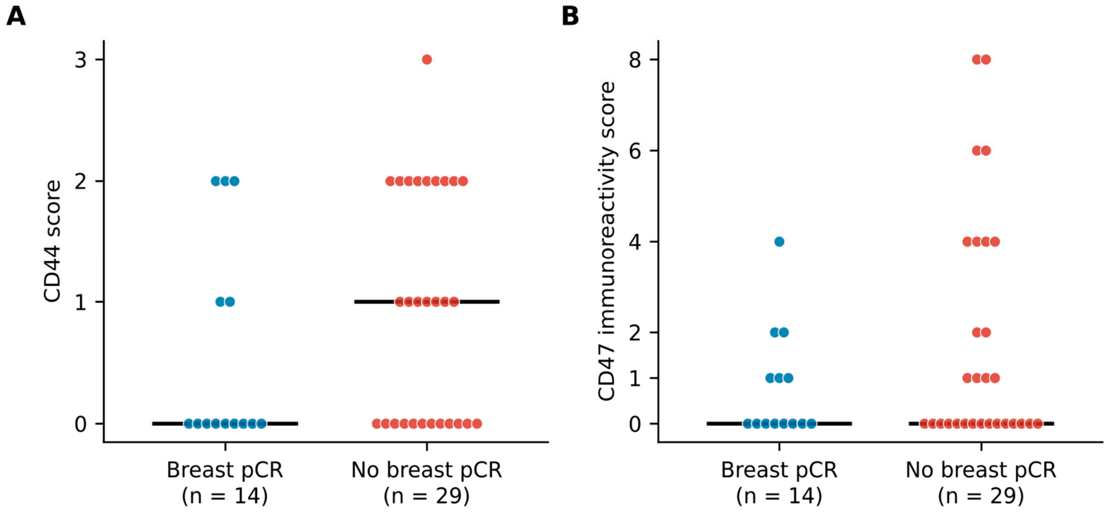

Figure S2. Individual pre-treatment marker scores according to breast pathological complete response (ypT0/is). (A) CD44 score; (B) CD47 immunoreactivity score. Each point represents one patient; points with the same score are spread horizontally to reduce overlap, and horizontal bars indicate medians. pCR, pathological complete response.

Table S1. Univariable logistic regression for total and breast pathological complete response (n = 43): standard maximum likelihood and Firth penalized likelihood estimates.

	Total pCR (ypT0/is ypN0)				Breast pCR (ypT0/is)			
**Variable**	**Standard OR (95% CI)**	**P**	**Firth OR (95% CI)**	**P**	**Standard OR (95% CI)**	**P**	**Firth OR (95% CI)**	**P**
CD44 score, per 1-point increase	0.53 (0.23–1.24)	0.143	0.57 (0.24–1.20)	0.145	0.60 (0.28–1.29)	0.192	0.63 (0.29–1.27)	0.201
CD44 positive vs. negative	0.36 (0.09–1.46)	0.153	0.39 (0.09–1.43)	0.156	0.39 (0.10–1.47)	0.164	0.41 (0.11–1.45)	0.169
CD47 IRS, per 1-point increase	0.80 (0.54–1.19)	0.264	0.84 (0.54–1.14)	0.299	0.77 (0.52–1.13)	0.185	0.81 (0.53–1.10)	0.192
CD47 IRS > 0 vs. 0 [a]	0.76 (0.20–2.93)	0.692	0.78 (0.20–2.85)	0.708	0.80 (0.22–2.90)	0.739	0.82 (0.23–2.84)	0.751
CD47 IRS ≥ 4 vs. 0–2 [a]	0.26 (0.03–2.36)	0.232	0.36 (0.04–1.92)	0.249	0.20 (0.02–1.81)	0.152	0.28 (0.03–1.47)	0.142
CD47 IRS ≥ 7 vs. 0–6 (prespecified)	NE		0.47 (<0.01–6.37)	0.611	NE		0.38 (<0.01–5.08)	0.503
CD47 IRS 1–2 vs. 0 [a]	1.31 (0.29–5.95)	0.730	1.32 (0.29–5.65)	0.709	1.56 (0.36–6.76)	0.550	1.54 (0.37–6.44)	0.547
CD47 IRS ≥ 4 vs. 0 [a]	0.29 (0.03–2.74)	0.277	0.39 (0.04–2.25)	0.310	0.23 (0.02–2.22)	0.206	0.32 (0.03–1.83)	0.215
CD47 three-level variable, overall test [a]	—	0.377	—	0.463	—	0.211	—	0.275

Standard estimates: Wald 95% confidence intervals and P values; for the three-level variable, likelihood ratio test. Firth estimates: profile penalized likelihood 95% confidence intervals and penalized likelihood ratio tests. [a] Defined post hoc. NE, not estimable because of complete separation (both tumors with IRS ≥ 7 had residual disease). OR, odds ratio; CI, confidence interval; IRS, immunoreactivity score; pCR, pathological complete response.

Table S2. Pre-treatment CD44 and CD47 expression according to breast pathological complete response (ypT0/is; n = 43).

	**Breast pCR (n = 14), n (%)**	**No breast pCR (n = 29), n (%)**	**Breast pCR rate, % (95% CI)**	**P**
CD44 score, median (IQR)	0 (0–1)	1 (0–2)	—	0.187 [a]
0	9 (64.3)	12 (41.4)	42.9 (21.8–66.0)	
1	2 (14.3)	7 (24.1)	22.2 (2.8–60.0)	
2	3 (21.4)	9 (31.0)	25.0 (5.5–57.2)	
3	0 (0.0)	1 (3.4)	0.0 (0.0–97.5)	
CD47 IRS, median (IQR)	0 (0–1)	0 (0–4)	—	0.420 [a]
0	8 (57.1)	15 (51.7)	34.8 (16.4–57.3)	
1	3 (21.4)	4 (13.8)	42.9 (9.9–81.6)	
2	2 (14.3)	2 (6.9)	50.0 (6.8–93.2)	
4	1 (7.1)	4 (13.8)	20.0 (0.5–71.6)	
6	0 (0.0)	2 (6.9)	0.0 (0.0–84.2)	
8	0 (0.0)	2 (6.9)	0.0 (0.0–84.2)	
CD44 status				0.203 [b]
Negative (score 0)	9 (64.3)	12 (41.4)	42.9 (21.8–66.0)	
Positive (score ≥ 1)	5 (35.7)	17 (58.6)	22.7 (7.8–45.4)	
CD47, any expression [c]				1.00 [b]
IRS 0	8 (57.1)	15 (51.7)	34.8 (16.4–57.3)	
IRS > 0	6 (42.9)	14 (48.3)	30.0 (11.9–54.3)	
CD47, sensitivity threshold [c]				0.231 [b]
IRS 0–2	13 (92.9)	21 (72.4)	38.2 (22.2–56.4)	
IRS ≥ 4	1 (7.1)	8 (27.6)	11.1 (0.3–48.2)	
CD47, prespecified threshold				1.00 [b]
IRS 0–6	14 (100.0)	27 (93.1)	34.1 (20.1–50.6)	
IRS ≥ 7	0 (0.0)	2 (6.9)	0.0 (0.0–84.2)	

Percentages in the first two columns are calculated within response groups; breast pCR rates are calculated within marker categories, with Clopper–Pearson exact confidence intervals. [a] Mann–Whitney U test. [b] Fisher exact test. [c] Defined post hoc. pCR, pathological complete response; IRS, immunoreactivity score; IQR, interquartile range; CI, confidence interval.

Table S3. Pathological complete response and marker expression according to neoadjuvant treatment category (n = 43).

Group	n	Total pCR	Breast pCR	CD44+	CD47 IRS > 0	Total pCR, CD44–	Total pCR, CD44+	Total pCR, CD47 IRS 0	Total pCR, CD47 IRS > 0
Anthracycline yes	34	7/34	8/34	16/34	16/34	7/18	0/16	3/18	4/16
Anthracycline no	9	5/9	6/9	6/9	4/9	1/3	4/6	4/5	1/4
Taxane yes	41	12/41	14/41	21/41	20/41	8/20	4/21	7/21	5/20
Taxane no	2	0/2	0/2	1/2	0/2	0/1	0/1	0/2	—
Platinum yes	11	6/11	7/11	6/11	5/11	2/5	4/6	4/6	2/5
Platinum no	32	6/32	7/32	16/32	15/32	6/16	0/16	3/17	3/15
Trastuzumab yes	16	6/16	8/16	8/16	7/16	3/8	3/8	4/9	2/7
Trastuzumab no	27	6/27	6/27	14/27	13/27	5/13	1/14	3/14	3/13

Values are n/N. Treatment categories are not mutually exclusive and closely followed molecular subtype: all 22 patients with HR-positive/HER2-negative disease received an anthracycline (21 with a taxane) and none received platinum, and all 16 patients with HER2-positive disease received trastuzumab. No statistical testing was performed; —, not applicable (no patient in the category). pCR, pathological complete response (total, ypT0/is ypN0; breast, ypT0/is); IRS, immunoreactivity score.

Table S4. Sensitivity analysis of CD44 at a higher cut-off (score ≥ 2): pathological complete response (n = 43).

Endpoint	CD44 score ≥ 2, n/N (%; 95% CI)	CD44 score 0–1, n/N (%; 95% CI)	OR (95% CI)	P
Total pCR (ypT0/is ypN0)	2/13 (15.4; 1.9–45.4)	10/30 (33.3; 17.3–52.8)	0.36 (0.07–1.96)	0.290
Breast pCR (ypT0/is)	3/13 (23.1; 5.0–53.8)	11/30 (36.7; 19.9–56.1)	0.52 (0.12–2.30)	0.491

Defined post hoc. Score ≥ 2 corresponds to staining of more than 25% of tumor cells. Percentages are given with Clopper–Pearson exact 95% confidence intervals; odds ratios are from univariable logistic regression with Wald 95% confidence intervals; P values are from the Fisher exact test. pCR, pathological complete response; OR, odds ratio; CI, confidence interval.

Table S5. Sensitivity analysis of CD47 after exclusion of the five tumors with IRS 0 and no internal control staining.

Endpoint and analysis	All tumors (n = 43)	Five tumors excluded (n = 38)
**Total pCR (ypT0/is ypN0)**		
IRS > 0 vs. IRS 0 [a]	5/20 (25.0) vs. 7/23 (30.4); P = 0.745	5/20 (25.0) vs. 6/18 (33.3); P = 0.724
IRS ≥ 4 vs. IRS 0–2 [a]	1/9 (11.1) vs. 11/34 (32.4); P = 0.405	1/9 (11.1) vs. 10/29 (34.5); P = 0.237
IRS, per 1-point increase, OR (95% CI)	0.80 (0.54–1.19); P = 0.264	0.77 (0.51–1.17); P = 0.220
**Breast pCR (ypT0/is)**		
IRS > 0 vs. IRS 0 [a]	6/20 (30.0) vs. 8/23 (34.8); P = 1.00	6/20 (30.0) vs. 6/18 (33.3); P = 1.00
IRS ≥ 4 vs. IRS 0–2 [a]	1/9 (11.1) vs. 13/34 (38.2); P = 0.231	1/9 (11.1) vs. 11/29 (37.9); P = 0.223
IRS, per 1-point increase, OR (95% CI)	0.77 (0.52–1.13); P = 0.185	0.77 (0.51–1.15); P = 0.197

Values are n/N (%) of patients achieving pathological complete response in each marker category. The five excluded tumors had an IRS of 0 and no stained internal control element (erythrocytes, lymphocytes, or endothelial cells); one achieved total pCR and two achieved breast pCR. The sensitivity analysis was defined post hoc. P values for categorical comparisons are from the Fisher exact test; per-point odds ratios and P values are from univariable logistic regression with Wald 95% confidence intervals. [a] Defined post hoc. IRS, immunoreactivity score; pCR, pathological complete response; OR, odds ratio; CI, confidence interval.
